# 

**Published:** 1852-04

**Authors:** 


### NOTICE TO CORRESPONDENTS.

The case by *Medicus* will be published, if authenticated by the narrator's name.

Communications and Books for notice should be addressed to the Editors, care of Messrs. Lindsay & Blakiston.

Letters, &c., connected with the *business affairs* of the Journal should be addressed to the Publishers.

Papers for publication must be received *before* the 20th of the month, or they cannot appear in the forthcoming number.

The following Journals have been received in exchange:

New York Medical Times.
Medical News. Philadelphia.
New York Medical Gazette.
The Boston Medical and Surgical Journal. (Weekly.)
Stethoscope and Virginia Medical Gazette.
Western Journal.
Buffalo Journal.
Transylvania Medical Journal.
Nashville Journal of Medicine and Surgery.
Southern Medical and Surgical Journal.
New Hampshire Journal of Medicine.
Opal, from the Utica Insane Asylum.
Dental Times and Advertiser.
The London Lancet. (Weekly, London.)
The Medical Times. (Weekly, London.)
Dublin Medical Press.
Journal of Pharmacy.
North Western Medical Intelligencer.
American Journal of Insanity. Oct.
New Jersey Medical and Surgical Reporter.

---

### BOOKS RECEIVED.

Alin on Retro-pharyngeal Abscess.
Operative Surgery of Bernard and Huette, translated by Drs. Van Buren and Isaacs, New York.
Report of the Ohio Lunatic Asylum for the year 1851.
Outlines of the Arteries and Nerves, by J. Neill, M. D. From the Publishers.
Swett on Diseases of the Chest. From the Publishers.
Graham's Elements of Chemistry. From Blanchard & Lea.

---

Communications have been received from:—
Dr. L. Turnbull, Philadelphia.
Dr. F. W. Sargent, Philadelphia.
Dr. J. A. Meigs, Philadelphia.

---

The foreign correspondents of the Examiner will please direct their Exchanges and other communications to the care of Mr. Thos. Delf, No. 12 Paternoster Row, London, or Mr. H. Bossange, 21 Bis, Quai Voltaire, Paris.

